# Impact of the gut microbiome on the genome and epigenome of colon epithelial cells: contributions to colorectal cancer development

**DOI:** 10.1186/s13073-019-0621-2

**Published:** 2019-02-25

**Authors:** Jawara Allen, Cynthia L. Sears

**Affiliations:** 1 Department of Medicine, Johns Hopkins University School of Medicine, Orleans Street, Baltimore, MD 21231 USA; 2 Bloomberg-Kimmel Institute for Immunotherapy and Department of Oncology, Sidney Kimmel Comprehensive Cancer Center, Johns Hopkins Medical Institutions, North Broadway, Baltimore, MD 21231 USA

## Abstract

In recent years, the number of studies investigating the impact of the gut microbiome in colorectal cancer (CRC) has risen sharply. As a result, we now know that various microbes (and microbial communities) are found more frequently in the stool and mucosa of individuals with CRC than healthy controls, including in the primary tumors themselves, and even in distant metastases. We also know that these microbes induce tumors in various mouse models, but we know little about how they impact colon epithelial cells (CECs) directly, or about how these interactions might lead to modifications at the genetic and epigenetic levels that trigger and propagate tumor growth. Rates of CRC are increasing in younger individuals, and CRC remains the second most frequent cause of cancer-related deaths globally. Hence, a more in-depth understanding of the role that gut microbes play in CRC is needed. Here, we review recent advances in understanding the impact of gut microbes on the genome and epigenome of CECs, as it relates to CRC. Overall, numerous studies in the past few years have definitively shown that gut microbes exert distinct impacts on DNA damage, DNA methylation, chromatin structure and non-coding RNA expression in CECs. Some of the genes and pathways that are altered by gut microbes relate to CRC development, particularly those involved in cell proliferation and WNT signaling. We need to implement more standardized analysis strategies, collate data from multiple studies, and utilize CRC mouse models to better assess these effects, understand their functional relevance, and leverage this information to improve patient care.

## Background

Human gut microbiome composition has recently been associated with a myriad of diseases, ranging from autism and schizophrenia to inflammatory bowel disease and colorectal cancer (CRC) [1–3]. Many of the associations between the gut microbiome and disease implicate both the microbiome composition overall and specific microbial species in disease development; the link between the gut microbiome and cancer is no exception. Several studies show that both the overall gut microbiome composition and microbial organization differ in CRC patients compared to healthy individuals [4–9]. Other studies show that, in some cases, particular microbial species are present more frequently in tumor tissue than in flanking normal tissue throughout the entire progression of disease, from early tumor development to metastasis [5, 7, 10–22]. Table 1 summarizes key features of the major changes in the gut microbiome and the individual microbes associated with CRC.

**Table 1:** Overview of microbiome and specific microbe associations with colorectal cancer^a^

Microbiome or single microbes	CRC associations	CRC animal models	CRC pathways	Effects on the genome or epigenome
Microbiome composition	Results are variable but several groups of microbes are found more frequently in CRC patients than normal controls: microbes associated with periodontal disease [6], human oral microbes (*Fn*, *Pm*, *Ps*, and *Gm*) [7–9], *Klebsiella*, *Enterococcus*, *Escherichia/Shigella*, *Streptococcus*, and *Peptostreptococcus* [4]. Several species are found with increasing frequency as tumor stage progresses from healthy tissue to advanced adenoma (*Bd* and *Bm*) or from advanced adenoma to carcinoma (*Bo* and *Bv*) [5]	In an AOM mouse model of CRC, inoculation of mice with human gut microbial communities produces variable amounts of tumor formation associated with microbiome composition [23], and perhaps with donor CRC status [24]	Inoculation of mice with human gut microbial communities leads to increased expression of proinflammatory cytokines, increased expression of genes involved in proliferation, apoptosis, stemness, invasiveness and metastasis, and/or increased Th1 and Th17 cell populations [24]	Use of antibiotics and GF mice has previously suggested a role for gut microbe-induced methylation changes in specific genes and expression changes in miRNAs [131–135]
Microbiome organization	In humans, invasive polymicrobial bacterial biofilms are present more frequently on right-sided tumors than on left-sided tumors [3, 7]	Not yet identified	The presence of invasive polymicrobial bacterial biofilms in humans is associated with decreased E-cadherin protein detection, increased IL-6 protein expression, increased STAT3 activation, and increased cell proliferation in CECs [3]	Not yet identified
ETBF	ETBF is found more frequently in individuals with CRC than in healthy controls [11–13]	In an *Apc*^*min/+*^ mouse model of CRC, ETBF inoculation results in an IL-17-dependent increase in tumorigenesis in the distal colon [27]	Inoculation of mice with ETBF leads to a proinflammatory immune environment characterized by STAT3 activation, IL-17-dependent NF-κB activation, increased WNT/β-catenin signaling, E-cadherin cleavage, and increased CEC proliferation [29–31, 33]	In *Apc*^*min/+*^ mice, ETBF induces enrichment of EZH2 and DNMT1 at promoter CpG islands of specific genes in inflamed distal CECs [122]. BFT induces CEC DNA damage, possibly through the induction of spermine oxidase with generation of ROS [136]
*pks + Escherichia coli*	*pks + E. coli* are found more frequently in individuals with CRC than in healthy controls [14, 15], more frequently in tumors than in normal flanking tissue [14], and more frequently in late-stage tumors than in early-stage tumors [14]	In conventional and GF *Il10*^−/−^/AOM mouse models of CRC, *pks + E. coli* induce tumor formation [25, 26]	Not yet identified	*pks + E. coli* genotoxin colibactin crosslinks DNA, leading to dsDNA breaks and CIN [55, 56, 137]
*Fusobacterium nucleatum*	When compared to normal colon tissue, fusobacteria are found more frequently in adenoma samples [7, 17], colon tumor samples with high-grade dysplasia [20], carcinoma samples [16, 18], and even distant CRC metastases [19]. *Fn* was identified as the dominant species in many of these studies, although the impact of the four subspecies of *Fn* is uncertain	In a conventional *Apc*^*min/*+^ mouse model of CRC, *Fn* increases tumor formation, whereas in a GF *Il10*^*−/−*^ mouse model of CRC or a *T-bet*^*−/−*^/*Rag2*^*−/−*^mouse model of CRC, *Fn* has no effect on tumor formation [16]	Inoculation of mice with *Fn* leads to increased β-catenin signaling in CECs, increased cell proliferation, myeloid cell accumulation, and the induction of proinflammatory cytokines [32]	See text and Table 2
*Streptococcus gallolyticus*	*Sg* bacteremia is strongly associated with colon tumor presence. *Sg* is found more frequently in tumors than in surrounding normal tissue [21, 22]	In a mouse xenograft model of CRC, *Sg* promotes tumor growth. In an AOM mouse model of CRC, *Sg* promotes tumor development [28]	Inoculation of mice with *Sg* leads to increased β-catenin nuclear localization, and to increased expression of c-Myc and cyclin D1 proteins [28]	Not yet identified
*Enterococcus faecalis*	Not yet identified	*In an Il10*^*−/−*^mouse model, colonization with superoxide-producing *Ef* leads to cancer formation [138]	*Ef* activates WNT/β-catenin signaling and transcription factors associated with CRC stem cells through a bystander effect [139]	See text and Table 2

^a^This table provides a summary of the associations between various microbes or microbial communities and colorectal cancer (CRC). Epidemiologic data, in vivo animal experiments, and key pathways associated with CRC are presented. Also, specific genome or epigenome effects that do not fall within the scope of the articles reviewed (studies examining direct effects of gut microbes on CECs, 2015–present) are highlighted. Abbreviations: *AOM* azoxymethane, *Bd Bacteroides dorei*, *Bf Bacteroides fragilis*, *BFT Bacteroides fragilis* toxin, *Bm Bosea massiliensis*, *Bo Bacteroides ovatus*, *Bv Bacteroides vulgatus*, *CEC* colon epithelial cell, *CIN* chromosomal instability, *DNMT1* DNA methyltransferase 1, *dsDNA* double-stranded DNA, *Ef Enterococcus faecalis*, *ETBF* enterotoxigenic *Bacteroides fragilis*, *EZH2* Enhancer of Zeste protein-2, *Fn Fusobacterium nucleatum*, *Gm Gemella morbillorum*, *Pm Parvimonas micra*, *Ps Peptostreptococcus stomatis*, *ROS* reactive oxygen species, *Sg Streptococcus gallolyticus*, *STAT3* signal transducer and activator of transcription 3

In an effort to move past correlation into the realm of causation, various microbial communities, and individual microbes, have been tested for their abilities to induce tumor formation in mouse models of CRC. As outlined in Table 1, some studies have used azoxymethane (AOM), a carcinogenic compound that induces colonic epithelial cell (CEC) mutations (largely affecting the WNT pathway), to test whether specific microbial communities impact colon tumorigenesis in mice [23–26]. Other studies have utilized *Apc*^*min/+*^ mice, a genetic model of CRC in which mice are predisposed to intestinal adenoma formation as a result of a mutation in the *Apc* gene and increased WNT signaling [16, 27, 28]. These models facilitate dissection of the CEC pathways that are altered by the gut microbiome, and have been used to identify microbe-induced changes in WNT signaling, β-catenin nuclear localization, IL-6 expression, STAT3 activation, E-cadherin cleavage, cell proliferation, inflammation, and immune cell infiltration [27–33]. To date, we have strong evidence that both microbial community composition and organization and the presence of specific microbes are associated with various stages of CRC development, and that these microbes could initiate tumor formation and contribute to tumor growth in vivo.

Nevertheless, cancer is a disease that is initiated and progresses (via processes including tissue invasion and metastasis) through changes in the genome and epigenome [34, 35]. So, to establish a direct, causal connection between the gut microbiome and CRC development, we must determine whether and how microbes alter mutation rates, gene methylation, chromatin structure, and/or non-coding RNA expression in CECs. Several epidemiological studies have associated specific bacteria in the gut with tumors that are characterized by DNA hypermethylation [36–39] or by specific mutational patterns [40], strengthening the hypothesis that gut microbes have a role in CRC development through their effects on the genome and epigenome of CECs.

Gut microbes could elicit their effects on the genome or epigenome via direct or indirect mechanisms. There are two key indirect mechanisms. The first is the capacity of gut microbes to induce a pro-carcinogenic inflammatory response [41–43]. The second is the production of secondary metabolites by gut microbes [44–46]. The ability of short chain fatty acids (SCFAs), hydrogen sulfide (H_2_S), secondary bile acids, and many other metabolites to impact the genome or epigenome of CECs, to alter rates of CRC progression, and to function as targets for CRC prevention or treatment is tremendously important and has thus been the topic of many recent reviews [47–50]. Overall, SCFAs (such as acetate, propionate, and butyrate) have been shown to function in the suppression of inflammation—for example, downregulation of pro-inflammatory cytokines and induction of FOXP3^+^ T regulatory cell differentiation—and thus are thought to possess mostly anti-carcinogenic properties. By contrast, H_2_S, secondary bile acids, and other metabolites have been shown to cause DNA damage, and thus are thought to be more pro-carcinogenic [50].

In this review, we focus on examining recent articles (2015–present) that describe the direct effects of bacteria on CECs. We highlight studies that have utilized live bacteria, bacterial communities, or species-specific virulence factors to determine whether microbes can alter the genome or epigenome in ways that directly propel CEC transformation and the clonal expansion that defines CRC (Table 2). We also discuss recent studies in which direct effects of microbial metabolites on the genome or epigenome of CECs have been demonstrated. We anticipate that a more complete understanding of all of these effects will allow us to add microbiome data to the accruing CEC genetic and epigenetic data used to screen for CRC. Moreover, we predict that these data will enable the development of combination strategies for the prevention and treatment of CRC that target: (i) CEC pathways that are altered by genome or epigenome changes; and (ii) the microbiome, for example, via bacteriophage microbiome modulation, targeted antibiotics, and/or specific bacterial vaccines.

**Table 2:** Summary of recent papers (2015–present) addressing the impact of gut microbes on the colon epithelial cell genome or epigenome

Key findings	Bacteria studied	Model system	Relevant CRC genes or pathways identified	Technique used to examine genome or epigenome
DNA damage				
Colibactin-induced intrastrand DNA crosslinking upon exposure to cells [57]	*pks + E. coli*	HeLa cells	None highlighted	Cross-linking assay in cells, acellular DNA-cross-linking assay
*pks + E. coli* worked synergistically with ETBF to cause increased DNA damage and increased tumor formation in a mouse model of CRC [59]	*pks + E. coli* and *ETBF*	Mice	None highlighted	Immunohistochemistry
When inoculated with ETBF, *Apc*^min/+^/*Msh*2^−/−^ mice produced more tumors than *Apc*^min/+^ mice. The increase in tumor burden was not seen in the absence of ETBF inoculation, suggesting that MMR proteins are important in preventing tumorigenesis after ETBF infection [61]	ETBF	Mice	None highlighted	Transgenic mouse model
CECs exposed to macrophages that were previously exposed to *E. faecalis* showed an increased rate of mutagenesis and an increased rate of aneuploidy and chromosomal translocation, indicative of CIN [63]	*E. faecalis*	Young adult mouse colonic (YAMC) ECs	Several cancer driver genes, including *Arid1b*, *Cdkn2a*, *Daxx*, *Gata3*, *Map3k1*, *Notch1*, *Pten*, *Smad2* and others	Mutant fraction assay, FACS
Methylation				
In a porcine model, in which premature infant pigs were given antibiotics immediately after birth, 80 DMRs were identified and were associated with genes involved in phagocytosis, the innate immune response, and other pathways [73]	Antibiotic-treated porcine gut microbiome	Premature infant pigs	Pathways related to innate immune response and phagocytosis	RRBS, BSP
Treatment of human intestinal ECs with *Lactobacillus acidophilus*, *Bifidobacterium infantis*, and *Klebsiella* species resulted in methylation changes in several hundred genes of interest [74]	*L. acidophilus*, *B. infantis and Klebsiella* species	Human intestinal EC lines (H4 and NCM460)	Pathways related to nucleotide binding (immature ECs) and chromatin organization (mature ECs)	Infinium Human Methylation 450 BeadChip
Fecal microbial transplant to reintroduce microbes into GF mice resulted in increased gene methylation [75]	Murine gut microbiome	Mice	None highlighted	Bisulfite pyrosequencing
The gene methylation status of GF mice differed from that of conventional mice. The number of genes with changes in both gene expression and methylation status increased as mice aged [76]	Murine gut microbiome	Mice	Pathways related to cellular proliferation or regeneration (*Pik3cd*, *Rb1*, *Grb10*, *Plagl1*, *Nfix*, *Tab3*) and immune response (*Atp7a*, *Atf4*, *Bcl3*)	RRBS
ETBF-induced tumors contained more hypermethylated DMRs and fewer hypomethylated DMRs than spontaneous tumors [61]	ETBF	Mice	*Hoxa5*, *Polg*, *Runx1*, *Runx3*, *CD37*, *Stx11*, *Tceb2*, *Lgr6*, *Cdx1*, and *Fut4* genes	MBD-seq, pyrosequencing, qMSP
Chromatin structure				
Investigators found no differential DNase hypersensitivity sites in the jejunum of GF mice. They did, however, find changes in the histone marks H3K4me1 and H3K27ac, which are generally enriched at poised or active enhancers, respectively [87]	Murine gut microbiome	Mice	Transcription factors belonging to the IRF family, STAT family, and ETS family	DNase-seq, ChIP-seq
Several hundred promoters and enhancers lost rhythmicity after antibiotic treatment, and a near equal number gained de novo rhythmic behavior [92]	Antibiotic-treated murine gut microbiome	Mice	None highlighted	ChIP-seq
Bacterial presence resulted in numerous changes in histone acetylation and methylation in the proximal colon tissue of GF mice. SCFAs produced by gut microbes might have mediated this effect [93]	Murine gut microbiome	Mice	None highlighted	Electrospray ionization tandem mass spectrometry
The location of H3K4 methylation marks was modified when gut microbes colonized GF mice [94]	Murine gut microbiome	Mice	Genes involved in maintaining the innate mucosal barrier, ROS generation, ephrin signaling, and others	ChIP-seq
In mice treated with antibiotics for 3 days, H3K18 crotonylation in the colon was decreased. This was associated with a concomitant decrease in HDAC2 protein expression, which was mediated by the SCFAs butyrate and crotonate. These SCFAs promote H3K18 crotonylation by inhibiting HDACs [96]	Antibiotic-treated murine gut microbiome	Mice, mouse small intestinal enteroids, human CRC cell lines (HCT116)	Pathways related to endometrial cancer, prostate cancer, pancreatic cancer, CRC, TGF-β signaling and stem cell pluripotency	ChIP-seq
Non-coding RNAs				
Using GF mice, the presence of gut microbes was associated with decreased production of miRNAs, which were shown to be produced by intestinal ECs, goblet cells and Paneth cells [107]	Murine gut microbiome	Mice	None highlighted	NanoString nCounter
Absence (GF mice) of the gut microbiota resulted in lower levels of expression of the miRNAs *let-7b*, *miR-141*, and *miR-200a.* Depletion (antibiotic-treated rats) of gut microbiota resulted in lower levels of miRNAs *let-7b*, *miR-141*, *miR-200a*, and *miR-1224* after 6 weeks of treatment [108]	Murine gut microbiome, antibiotic-treated murine gut microbiome	Mice, rats	*let-7b*, *miR-141*, *miR-200a*, and *miR-1224*	qRT-PCR
*miR-21-5p* was expressed at higher levels in the small and large intestine of conventional mice than in GF mice. Exposing HT-29 and SW480 cells (two CRC cell lines) to *Bacteroides acidifaciens* type A43 and *Lactobacillus johnsonii* 129 resulted in an upregulation of *miR-21-5p* [112]	Murine gut microbiome, *B. acidifaciens* type A43 and *L. johnsonii* 129	Mice, human CRC cell lines (HT-29, SW480)	*miR-21-5p*	Microarray
19 miRNAs were differentially expressed in IESCs of GF mice when compared to conventionalized mice. *miR-375-3p* was downregulated in conventionalized mice [115]	Murine gut microbiome	Mice, mouse small intestinal enteroids	*miR-375-3p*	RNA-seq
Several miRNAs were downregulated in *Fusobacterium nucleatum-*rich tumor samples from patients with recurrent CRC. A CRC xenograft model was used to show that *F. nucleatum* causes resistance to oxaliplatin and 5-FU via downregulation of *miR-4802* and miR-18a* [117]	*F. nucleatum*	Mice, CRC cell lines (HCT116 and HT29)	*miR-4802* (newly associated with CRC) and miR-18a* (belongs to the *miR-17-92* cluster)	RNA-seq
lncRNAs in the mouse duodenum, jejunum, ileum, and colon were altered in GF mice when compared to conventional mice [118]	Murine gut microbiome	Mice	Pathways related to GPCR signaling and TGF signaling	RNA-seq
When GF mice were reconstituted with normal mouse microbiota or with *E. coli* alone, fairly distinct changes in lncRNA signatures occurred, with only 8% of the differentially expressed lncRNAs overlapping [119]	Murine gut microbiome, *E. coli*	Mice	None highlighted	Affymetrix mouse exon microarray

Abbreviations: *BSP* bisulfite sequencing PCR, *CEC* colon epithelial cell, *ChIP* chromatin immunoprecipitation, *CIN* chromosomal instability, *DMR* differentially methylated region, *ETBF* enterotoxigenic *Bacteroides fragilis*, *EC* epithelial cell, *ETS* e26 transformation specific, *GF* germ-free, *FACS* fluorescence-activated cell sorting, *GPCR* G-protein-coupled receptor, *HDAC* histone deacetylase, *IESC* intestinal epithelial stem cell, *IRF* interferon regulatory factor, *lncRNA* long non-coding RNA, *MBD* methyl CpG binding domain, *miRNA* microRNA, *MMR* mismatch repair, *qMSP* quantitative methylation-specific PCR, *ROS* reactive oxygen species, *RRBS* reduced representation bisulfite sequencing, *SCFA* short chain fatty acid, *STAT* signal transducer and activator of transcription, *YAMC* young adult mouse colonic

## The genome

### The gut microbiome and DNA damage

The majority of spontaneous CRC development follows Knudson’s classic two-hit hypothesis [51, 52]. In this model, one mutation in each allele of the *APC* gene is needed to initiate tumorigenesis in the colon, and subsequent mutations in additional genes increase the rate of tumor growth and development [53, 54]. This pattern is seen in both hereditary and spontaneous CRC development, with at least 70–80% of spontaneous CRC tumors possessing mutations in both *APC* alleles [52]. As a result, when examining the impact of gut microbes on CRC development, it is important to determine whether the direct interaction between microbes and CECs can lead not only to DNA damage but also to specific gene mutations that contribute to CRC development.

*pks* + *Escherichia coli* are among the most extensively studied genotoxin-producing bacteria. They produce a cyclomodulin toxin called colibactin, which causes DNA double-strand breaks, chromosomal aberrations, and cell cycle arrest in cells in vitro [55, 56]. Recent studies have delved deeper into colibactin’s mechanism of action and have shown that, upon exposure to cells, this genotoxin induces intrastrand DNA crosslinking [57]. This crosslinking is accompanied by a robust ATR-dependent replication stress response [57], in which ATR phosphorylates many proteins that regulate origin of replication firing, cell cycle transitions, and replication fork progression [58]. This response prevents cells with damaged DNA from entering mitosis. In studies conducted by Dejea and colleagues [59], *pks* + *E. coli* were found to work synergistically with enterotoxigenic *Bacteroides fragilis* (ETBF) to cause increased DNA damage and increased tumor formation in a mouse model of CRC. This DNA damage was accompanied by a heightened inflammatory response that was necessary, but not sufficient, for increased colon tumor formation. The increased tumorigenesis was also highly dependent on the presence of both colibactin and *B. fragilis* toxin (BFT). Together, this evidence points to a direct correlation between these bacterial toxins, an increased inflammatory response, DNA damage, and tumor formation, but no studies to date have determined whether colibactin or BFT directly induces disease-initiating or disease-promoting DNA mutations in CECs.

Studies conducted using *E. coli* and ETBF provide clues as to how we can begin to dissect out the effects of DNA damage caused by their secreted toxins. In one study, Maddocks and colleagues [60] showed that enteropathogenic *E. coli* (EPEC) deplete the mismatch repair proteins of host cells, leading to an increased mutation frequency, as measured using an artificially inserted microsatellite. The effect was mediated by an EPEC-secreted protein (EspF) that targets the mitochondria of CECs and induces post-translational modifications of mismatch repair proteins [60]. In another study, Maiuri and colleagues [61] showed that, when inoculated with ETBF, *Apc*^*min/+*^*/Msh2*^*−/−*^ mice produced more tumors than *Apc*^*min/+*^ mice with intact Msh2 mismatch repair proteins. The increase in tumor burden was not seen in the absence of ETBF inoculation, suggesting that mismatch repair proteins play an important role in preventing tumorigenesis after ETBF colonization [61]. These approaches can be modified and used in vitro to determine whether bacterial toxins such as BFT and colibactin can directly cause DNA mutations in CECs. These methods only identify mismatch-repair-based increases in mutation rates, but other more generalized strategies are also available. The hypoxanthine phosphoribosyltransferase (HPRT)-forward mutation assay can be used to test the general mutation rate that is induced by a given compound. In this assay, the cells that are used contain one copy of the *HPRT1* gene. When grown in the presence of 6-thioguanine (6-TG), only cells that have acquired a mutation in their *HPRT1* gene are able to survive. So, by counting the number of cells that are alive after 6-TG treatment and comparing it to untreated controls, a general mutation frequency can be determined [62]. A similar assay was used by Wang and colleagues [63] to show that macrophages that are exposed to *Enterococcus faecalis-*induced mutations in a mouse colonic epithelial cell line.

Chromosomal instability (CIN) in epithelial cells is another mechanism that contributes to tumor formation. CIN has been identified in nearly all cancers, including CRC [53, 64, 65]. In order to determine whether bacteria can induce CIN in epithelial cells, immune cells have been used as an intermediary. Specifically, Wang and colleagues [63] first cultured macrophages in the presence of *E. faecalis*. They then exposed CECs to those macrophages and found an increased rate of aneuploidy and chromosomal translocation, indicative of CIN. These CECs were subsequently injected into the flank of NOD/SCID mice, which lack functioning T cells, B cells, and NK cells, and only CECs that had been exposed to the macrophages or a control carcinogen formed a tumor mass. Gene expression profiling of these masses revealed altered gene expression of at least three ‘driver genes’ in each sample [63]. This study highlights a novel microbial–macrophage interaction that induces pro-carcinogenic genome changes. Although these studies do not demonstrate direct effects of bacteria that lead to CIN in CECs, they do outline a methodology for future experiments; bacteria such as *pks* + *E. coli*, ETBF, and *Fusobacterium nucleatum* could be exposed to CECs and the cells could then be analyzed for chromosomal translocations and aneuploidy.

Whole-genome sequencing can also be used to measure mutation frequency and to observe pathogen-specific mutational patterns directly. In a study conducted by Szikriszt and colleagues [66], cisplatin treatment of a chicken lymphoblastic cell line was shown to induce primarily C > A mutations, a pattern found frequently in aflatoxin-induced cancers [66]. Importantly, the specific signature identified after cisplatin exposure differed when human cell lines were used (C > T instead of C > A mutations were most frequent) [67], which emphasizes the importance of relevant model selection in experimental design. These experiments would be particularly informative in models where bacterial communities, such as biofilms, induce tumor formation, as the causal bacteria are difficult to identify. Knowing the mutational signature caused by the biofilm may narrow down the list of driver organisms and provide us with a new target for screening.

## The epigenome

### The gut microbiome and DNA methylation

DNA methylation generally describes the addition of a methyl group (CH_3_) to a cytosine residue that precedes a guanine residue in DNA (termed CpG islands, often at or near the start site of gene transcription) [68]. The effects of DNA methylation on cancer development have been examined extensively. Two of the first studies showed both global and gene-specific DNA hypomethylation in cancer [69, 70]. Both hypomethylation and hypermethylation have been linked to CRC development, but the mechanisms by which they contribute to cancer development differ. DNA hypomethylation is generally thought to lead to tumorigenesis via one of three pathways: chromosomal instability, loss of imprinting, or reactivation of transposable elements [71]. Hypermethylation, on the other hand, is believed to lead to the decreased expression of tumor suppressor genes. Since the early studies, data have accrued to show that methylation differences play a major role in the initiation and progression of many types of cancer [72]. Much of this research has focused on CRC, where CpG island hypermethylation of *MLH1*, *RARB2*, *CDKN2A*, and other genes has been linked to tumor formation and growth [68, 71].

The question has been raised as to whether the gut microbiota are among the stimuli that can alter the balance of DNA methylation in CECs, and thus represent an avenue of investigation to determine whether there is a relationship between gut microbes, gene methylation, and the development of CRC. Two recent studies, using non-mouse models, have investigated this question. Pan and colleagues [73] used a porcine model, in which premature infant pigs were given antibiotics immediately after birth, to investigate the effect of early bacterial colonization in the gut on gene methylation. They found more than 80 differentially methylated regions (DMRs) in the distal small intestine and associated these regions with genes involved in phagocytosis, the innate immune response, and other pathways. Cortese and colleagues [74] used mature or immature human intestinal epithelial cell lines to investigate the impact of specific microbes on gene methylation status. This study showed that treatment of these cells with probiotic species (*Lactobacillus acidophilus* and *Bifidobacterium infantis*) or *Klebsiella* species resulted in methylation changes in several hundred genes of interest [74]. In immature epithelial cells, the common differentially methylated genes belonged to nucleotide-binding pathways, whereas in mature cells, the common differentially methylated genes belonged to chromatin organization pathways. Importantly, the majority of changes were specific to the bacteria used [74].

Other studies have looked to mice in order to tease out the relationship between the gut microbiome and CEC gene methylation status. Yu and colleagues [75] found that the presence of gut microbes led to an increase in the 3′ CpG island methylation of specific genes, which correlated with increased gene expression, suggesting a functional role for these changes. This result was corroborated when germ-free mice were conventionalized using fecal microbial transplants and the 3′ CpG island methylation status of two genes (*B4galnt1* and *Phospho1*) was examined [75]. A similar study showed that the methylation status of the CECs of germ-free mice differed from that in conventional mice, and that many of the affected genes are frequently mutated in CRC [76]. For example, the proto-oncogene *Bcl3* was hypomethylated and showed increased gene expression in conventional mice, whereas the tumor suppressor gene *Rb1* showed decreased gene expression in conventional mice. Although the difference in methylation status between germ-free mice and conventional mice seemed to wane as the mice aged, the number of genes with changes in both gene expression and methylation status increased as the mice aged, suggesting a decreased overall effect of gut microbes on gene methylation with time, but perhaps an increased functional effect [76].

The studies discussed so far have all examined the effects of microbes on methylation in normal CECs, but they did not examine these changes in transformed cells. One recent study has begun to address this knowledge gap. Maiuri and colleagues [61] compared the methylation profile of spontaneous tumors and ETBF-induced tumors in the distal colon of *Apc*^*min/+*^ mice. They found that ETBF-induced tumors contained more hypermethylated DMRs and fewer hypomethylated DMRs than spontaneous tumors. Furthermore, many of the hypermethylated DMRs were associated with the CpG islands of genes with known tumor-suppressive functions, such as *Hoxa5*, *Polg*, *Runx1*, *Runx3*, *CD37*, *Stx11*, *Tceb2*, *Lgr6*, *Cdx1*, and *Fut4* [61]. The expression of several of these genes was also reduced, but whether BFT induced these changes directly through interaction with CECs or indirectly via induced mucosal immune responses was not determined.

More studies are needed to better understand how methylation changes that are induced by specific microbes and their toxins contribute to CRC development. Initial experiments should focus on determining whether presumably health-promoting probiotic species, such as *Lactobacillus acidophilus*, have a common impact on methylation in CECs that is distinct from the signature induced by pathogenic bacteria. Furthermore, several studies have shown that butyrate can affect both the methylation of DNA globally [77, 78] and the expression of genes that function in DNA methylation or demethylation pathways [79, 80]. Because most of these experiments have been conducted in vitro using non-CEC lines and have only examined the effects of butyrate in isolation, in vivo studies should be conducted to determine whether butyrate-producing gut microbes can alter DNA methylation in CECs. Finally, more focus should also be placed on effects that are induced by specific bacterial toxins as strategies to detect, alter, or induce protective immunity to these toxins can be utilized more readily in the clinic.

### The gut microbiome and chromatin structure

In the nucleus, DNA is wrapped around histones, which are protein complexes composed of eight subunits. Each histone is made up of two copies each of H2A, H2B, H3, and H4 subunits, and the DNA–histone complex is referred to as a nucleosome. In general, the nucleus can be divided into regions of heterochromatin (areas in which nucleosomes are packed tightly together) or euchromatin (areas in which nucleosomes are more loosely packed). Areas of heterochromatin tend to be less transcriptionally active whereas areas of euchromatin tend to be more transcriptionally active. The location of histones is tightly regulated by a number of proteins and enzymes that modify the histones or serve as docking sites for other proteins that recognize those modifications [81]. Histone modifications include the methylation, acetylation, or phosphorylation of various residues, among others. Each modification has a unique impact on chromatin structure. For example, the acetylation of histone lysine residues is involved in transcriptional regulation and DNA repair. Histone acetylation and deacetylation are regulated by histone acetyltransferases, which acetylate histones, and histone deacetylases (HDACs), which remove acetyl groups from histones, respectively. Mutations in enzymes that belong to each of these groups have been found in cancer. HDAC inhibitors have already been approved for the treatment of hematologic malignancies, and growing evidence suggests they might be useful in CRC too [81, 82].

Much of the research surrounding the gut microbiome, CRC, and chromatin has focused on the role of butyrate as a HDAC inhibitor. The impact of butyrate has been explored in CRC in a number of studies, most of which show that it plays a protective role [47–49, 83], whereas other studies have supported a stimulatory role [84]. The most recent in vivo experiments to analyze the effects of butyrate on colon tumor formation used an AOM/dextran sodium sulfate (DSS) model of CRC to show that germ-free mice that were inoculated with the butyrate-producing bacterium *Butyrivibrio fibrisolvens* and given a high fiber diet were mostly protected from tumor formation. Importantly, mice given the bacterium alone or a high fiber diet alone were not protected, whereas mice given a mutant strain of *B. fibrisolvens* that produced lower levels of butyrate had intermediate protection from tumor formation [85]. Mechanistically, the tumors of mice given *B. fibrisolvens* and a high fiber diet had higher levels of histone subunit H3 acetylation, supporting the role of butyrate as a HDAC inhibitor. The role of butyrate in tumor formation and histone deacetylation has been well-studied and can be used to imply the potential impacts of the microbial community on histone deacetylation, but research on the direct effect of gut microbes on global chromatin structure and on the modulation of other histone marks is just beginning to pick up steam.

In an effort to expand our understanding of the effects of gut microbes on global chromatin structure, Camp and colleagues [86] examined the chromatin landscape of intestinal epithelial cells isolated from the ileum and colon of germ-free and conventionally reared mice. Surprisingly, using a modified DNase-seq hypersensitivity assay, they found no correlation between the presence of bacteria and chromatin accessibility. A more recent study looking at intestinal epithelial cells isolated from the jejunum of germ-free and conventional mice found similar results [87], suggesting rather definitively that gut microbes do not routinely induce changes in global chromatin accessibility. These results do not, however, rule out the potential impact of specific microbes or microbial communities on chromatin structure locally. Indeed, more site-specific analyses, performed by both Camp et al. [86] and Davison et al. [87], revealed greater accessibility of specific transcription factor binding sites in conventional mice. Both groups identified an upregulation in the accessibility of binding sites for transcription factors in the STAT (signal transducer and activator of transcription), IRF (interferon regulatory factor), and ETS (e26 transformation specific) families, each of which has been implicated in CRC progression [88–90]. Furthermore, many of these transcription factors were also identified by Richards and colleagues [91] as being differentially expressed after co-culture of CECs with gut bacteria. Taken together, these studies suggest that microbes alter the chromatin structure in specific regions, and that these changes have a large impact on the expression of genes that are known to be dysregulated in CRC.

Other studies examining the impact of the gut microbiome on chromatin structure in mice have investigated specific histone modifications. By assaying the location of multiple histone modifications using ChIP-Seq after the antibiotic treatment of mice, Thaiss and colleagues [92] showed that several hundred host gene promoters and enhancers lost rhythmicity following antibiotic treatment, and that a near equal number gained de novo rhythmic behavior. In other words, some mouse genes that display a diurnal pattern of promoter or enhancer chromatin structure no longer displayed this pattern upon antibiotic treatment. The relationship between these changes and CRC is uncertain, but as hundreds of genes were changed, these data need to be mined to determine whether the gut-microbiome-sensitive rhythmic changes in chromatin structure are related to CRC or other diseases. Krautkramer and colleagues [93] examined the proximal colon tissue of germ-free and conventional mice, and found that bacterial presence resulted in numerous changes in histone acetylation and methylation, but direct effects on CECs were not examined. For example, the amount of single acetylated lysine on histone subunit H3 was elevated in the proximal colon tissue of germ-free mice compared to conventional mice, whereas the amount of double acetylated lysine was reduced [93]. Furthermore, supplementation of the germ-free mouse diet with several SCFAs (acetate, propionate, and butyrate) resulted in a histone profile that more closely resembled that of conventional mice, suggesting that these metabolic byproducts of gut microbes induce histone modifications [93]. The functional implications of these changes in histone profile were assessed by examining gene-expression changes in the hepatocytes of germ-free and conventional mice. As expected, the identified pathways mostly related to metabolism. In future experiments, gene expression in CECs should be examined to determine whether these histone profile changes might contribute to CRC development.

Kelly and colleagues [94] also recently identified a connection between the gut microbiome and certain histone modifications. Specifically, the location of histones with a H3K4 methylation mark was shown to be modified by the presence of gut microbes. Because the location of histone H3 subunits was analyzed along with the presence or absence of K4 methylation marks, the authors were able to associate the changes with specific genes. This analysis revealed an abundance of genes that belonged to pathways associated with inflammatory bowel disease. Importantly, many of these genes and pathways are also associated with cancer (that is, genes involved in maintaining the innate mucosal barrier, reactive oxygen species generation, or ephrin signaling), so although the authors did not highlight a link to cancer in their findings, their results can be readily applied to better understand how gut microbes affect histone methylation at genes that are known to be dysregulated in CRC [94].

More novel histone modifications have also been associated with gut microbes. Histone crotonylation is the addition of crotonyl groups to a lysine residue of a histone subunit [95]. Crotonylation on lysine 18 of the histone subunit H3 (H3K18cr) is a common histone mark in the colon. Moreover, increased crotonylation at H3K18 is associated with the increased expression of genes that are linked to multiple cancers, including CRC [96]. H3K18 crotonylation in the colon decreased in mice treated with antibiotics for three days. This decrease was associated with a concomitant decrease in SCFAs and HDAC2 protein expression. Subsequent experiments showed that the SCFAs butyrate and crotonate promoted H3K18 crotonylation by inhibiting HDACs [96].

As the number of known post-translational histone modifications continues to increase [95], these results suggest a burgeoning role for these modifications in gut microbiome–CRC interactions, and perhaps potential new targets for intervention. Moreover, mouse models that test the tumorigenic effect of gut microbes or microbial communities are being used extensively, and thus should be employed to determine whether microbe-induced changes in specific histone modifications or the accessibility of specific transcription factor binding sites affects CRC pathogenesis.

### The gut microbiome and non-coding RNAs

Non-coding RNAs (ncRNAs) are RNA molecules that are transcribed from DNA but not translated into protein. They are generally classified into two groups: small non-coding RNAs (snRNAs) and long non-coding RNAs (lncRNAs) [97]. The most commonly studied snRNAs are microRNAs (miRNAs), which are approximately 22 nucleotides long [98]. By contrast, lncRNAs are ncRNAs that are always greater than 200 nucleotides in length, although some are much larger. MicroRNAs regulate protein-coding gene expression by binding to the 3′ UTR of mRNA molecules, causing repressed translation and encouraging the degradation of target mRNAs [99]. By contrast, lncRNAs generally regulate protein-coding gene expression by one of several mechanisms (for example, by acting as a scaffold for histone-modifying complexes, inhibiting the binding of transcription factors by direct binding to the transcription factors themselves or to their DNA targets, directly binding RNA polymerase 2, or binding and sequestering miRNAs) [100].

Dysregulation of both miRNAs and lncRNAs has been associated with CRC. Early studies identified a correlation between increased expression of particular miRNAs and the proto-oncogene *c-Myc* [101]. More recent studies have shown that miRNAs can drive the transformation from adenoma to adenocarcinoma [102], and that the microRNA 17/92 cluster can regulate the expression of common CRC-associated genes, including *BCL3* and *PTEN* [103, 104]. Long ncRNAs, including *HOTAIR*, *CCAT*, *MALAT-1*, *H19*, and many others, have been associated with CRC development, invasion, and metastasis and with early diagnosis and prognosis [105]. Interestingly, most lncRNAs are also associated with other cancers, suggesting that their functions span several different pathways and cell types.

The gut microbiome has been shown to regulate the expression of protein-coding genes in CECs [91, 92, 106], so it is not unreasonable to think that the gut microbiome might also regulate the expression of ncRNAs. Most studies to date have used germ-free and conventional mice to determine how lncRNA and miRNA expression differs in the presence of gut microbes. Using NanoString technology to examine the fecal miRNA profile of germ-free mice, conventional mice, and antibiotic-treated mice, Liu and colleagues [107] showed that the presence of gut microbes was associated with decreased fecal miRNA expression, although specific miRNAs were not examined. Also working with stool samples, but utilizing qRT-PCR and a specific set of four miRNAs known to be expressed in intestinal epithelial cells, Moloney and colleagues [108] showed that conventional mice produced higher levels of three of the four miRNAs (*let-7b*, *miR-141*, and *miR-200a*) than germ-free mice. Interestingly, when they utilized an antibiotic-treated rat model, all four miRNAs showed lower levels of expression after 6 weeks of antibiotic treatment, but at 2 weeks, half were upregulated and half were downregulated, suggesting a temporal nature to the antibiotic effect on miRNA expression. The potential functional consequences of these changes were not examined and are difficult to predict as *let-7b* functions as an anti-oncomiRNA (miRNAs that inhibit proto-oncogenes) and *miR-141* and *miR-200a* function as oncomiRNAs in CRC [109–111].

By deleting Dicer, a protein that is required for miRNA processing, Liu and colleagues [107] showed that intestinal epithelial cells, goblet cells, and Paneth cells each contribute to miRNA production, whereas lymphocytes do not. Other studies have examined intestinal epithelial cells directly to ensure that the observed miRNA differences were caused by the effect of gut microbes on epithelial cells alone. Using microarray and qPCR data, Nakata and colleagues [112] showed that *miR-21-5p* is expressed at higher levels in the small and large intestines of conventional mice than in germ-free mice. They then went on to show that exposing HT-29 and SW480 cells (two CRC cell lines) to heat-killed *Bacteroides acidifaciens* type A43 and to *Lactobacillus johnsonii* 129 resulted in an upregulation of *miR-21-5p*, suggesting that molecules derived from these bacteria (and not live bacteria alone) can directly regulate the expression of this well-studied oncomiRNA [112]. Paradoxically, both of these bacteria are regarded as probiotic bacteria and not oncogenic [113, 114], again indicating the need for studies focused on functional outcomes. Peck and colleagues [115] took their analysis a step further by isolating various epithelial cell subtypes from jejunal tissue of germ-free mice and of germ-free mice reconstituted with gut microbes for 2 weeks (conventionalized mice). They identified 11 miRNAs that were differentially expressed when all intestinal epithelial cell types were combined, and 19 miRNAs that were differentially expressed only in intestinal epithelial stem cells (IESCs), the cell type that showed the greatest change in miRNA expression. Although the majority of miRNAs showed increased expression in conventionalized mice compared to germ-free mice, the most highly expressed miRNA in IESCs (*miR-375-3p*) showed decreased expression, and knockdown of this particular miRNA in enteroids resulted in increased cellular proliferation [115]. Interestingly, *miR-375-3p* is downregulated in CRC tissues [116]. Thus, to date, several studies have shown that gut microbes can alter the expression of miRNAs, particularly those that are implicated in CRC development, but few studies have demonstrated a functional impact of these expression changes on tumor development in CRC models.

Following up on that idea, Yu and colleagues [117] used global miRNA expression profiling to identify several miRNAs that were downregulated in *F. nucleatum*-rich tumor samples from patients with recurrent CRC. These authors then treated CRC cell lines with inhibitors of two of these miRNAs (*miR-4802* and miR-18a*) and were able to demonstrate increased resistance to two common chemotherapy drugs used to treat CRC, oxaliplatin and 5-FU. By contrast, transfection of the same cells with miRNAs *miR-4802* and miR-18a* resulted in decreased drug resistance. Finally, a CRC xenograft model was used to demonstrate that *F. nucleatum* causes resistance to oxaliplatin and 5-FU by downregulating *miR-4802* and miR-18a* [117]. This is the most systematic example yet of how gut microbes might interact with CEC miRNAs to modulate CRC progression, and it should be used as a model for the future investigation of other CRC-associated gut microbes and miRNAs.

Less is known about the interaction between lncRNAs and the gut microbiome, probably because of difficulties in identifying the function of most lncRNAs. In one study, Dempsey and colleagues [118] found that the expression of lncRNAs in the mouse duodenum, jejunum, ileum, and colon was altered in the absence of gut microbes. Most of the DNA sequences encoding these lncRNAs were located in intergenic regions or in the introns of protein-coding genes, and the lncRNAs were predicted to function in regulating the expression of those genes. In the colon specifically, genes related to transforming growth factor (TGF) signaling and G-protein-coupled receptor (GPCR) signaling were identified. Liang and colleagues [119] examined the change in lncRNA expression that occurs when germ-free mice are reconstituted with normal mouse microbiota or with *E. coli* alone. Interestingly, the two different types of microbiome reconstitution resulted in fairly distinct changes in lncRNA signatures with only 8% overlap (six lncRNAs). These six lncRNAs were not associated with genes, but the authors did note that they are highly expressed in the thymus and spleen, suggesting a potential role in immunity [119].

Notably, the study by Liang and colleagues [119] was conducted using a publicly available database of microarray data. This research strategy can be used to further the field of gut microbiome–lncRNA interactions by mining data from other RNA-sequencing studies that disregarded lncRNAs in their analysis, or that analyzed lncRNAs in conjunction with protein-coding genes. For example, Peck and colleagues [115] identified 1157 protein-coding genes and lncRNAs that were upregulated or downregulated in the IESCs of conventional mice when compared to those of germ-free mice [115]. The genes that were elevated in conventional mice were involved in processes such as ‘mitotic cell cycle’ and ‘nuclear division’, suggesting a role in cell proliferation and potentially CRC progression. These data should be further examined to determine whether lncRNAs show the same or a different pattern when analyzed alone.

## Mechanistic insights

**Fig. 1:**
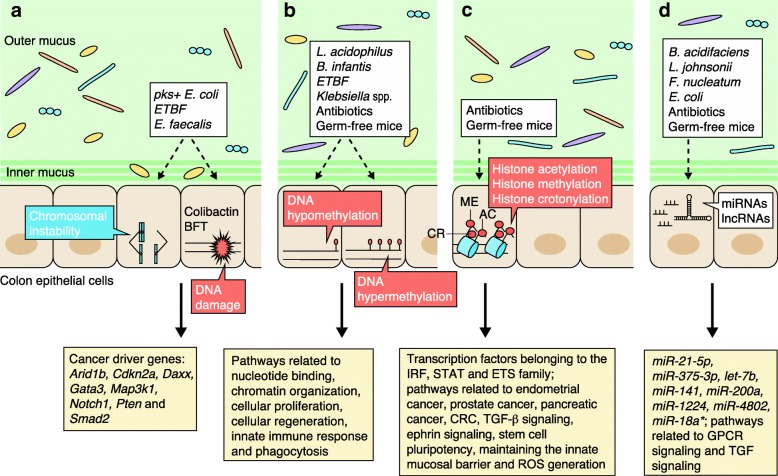
Effect of the gut microbiome on the colon epithelial cell genome and epigenome. **a** Enterotoxigenic *Bacteroides fragilis* (ETBF) and *pks + Escherichia coli* cause DNA damage in CECs that is mediated by *B. fragilis* toxin (BFT) and colibactin, respectively. *Enterococcus faecalis*, through impact on macrophages, induces chromosomal instability and tumor-inducing DNA mutations in cancer driver genes. **b** Antibiotics, germ-free mice, and specific microbes (*Bifidobacterium infantis*, *Lactobacillus acidophilus*, *Klebsiella* species, and ETBF) have been used to show that gut microbes induce both the hypermethylation and the hypomethylation of genes belonging to pathways that are dysregulated in colorectal cancer (CRC). **c** Antibiotics and germ-free mice have been used to show that gut microbes do not generally affect global chromatin structure in CECs, but do cause changes in the accessibility of transcription factor binding sites, in histone modifications, and in the location of those modified histones. These modifications often affect the promoter and enhancer regions of genes that belong to pathways that are dysregulated in CRC. **d** Antibiotics, germ-free mice, and specific microbes (*Bacteroides acidifaciens*, *Lactobacillus johnsonii*, and *Fusobacterium nucleatum*) have been used to show that gut microbes alter the expression of oncomiRNAs and anti-oncomiRNAs in CECs. They also alter the expression of long non-coding RNAs (lncRNAs) that are involved in G protein-coupled receptor (GPCR) and transforming growth factor (TGF) signaling. Abbreviations: *ETS* e26 transformation-specific, *IRF* interferon regulatory factor, *miRNA* microRNA, *ROS* reactive oxygen species, *STAT* signal transducer and activator of transcription

Figure 1 provides an overview of the mechanisms by which bacterial communities and species might impact the CEC genome or epigenome, thus altering tumor initiation, growth, and metastasis. An understanding of these mechanisms is necessary to develop creative approaches for the prevention, detection, and treatment of CRC. Most studies to date have examined the effects of changing the microbial community by using either antibiotics or germ-free mice, but only a few have studied the effects of specific bacteria. These studies show that altering the microbial community has a large impact on DNA methylation, histone modifications, and ncRNA expression patterns. The effects on broad categories of genes, such as those involved in cell proliferation, WNT signaling, maintenance of the innate mucosal barrier, generation of reactive oxygen species, ephrin signaling, or TGF-β signaling, have been shown by several groups.

Moreover, the patterns of methylation and promoter or enhancer histone marks in genes that are often dysregulated in CRC (such as *Arid1b*, *Cdkn2a*, *Daxx*, *Gata3*, *Map3k1*, *Notch1*, *Pten*, *Smad2*, *Hoxa5*, *Polg*, *Runx1*, *Runx3*, *CD37*, *Stx11*, *Tceb2*, *Lgr6*, *Cdx1*, and *Fut4*) and the expression of miRNAs such as *miR-375-3p*, *miR-21*, *miR-182*, and *miR-503* have been shown to be modulated by the gut microbiome. It is tempting to link changes in the gut microbiome to CRC-related pathways exclusively, but it is imperative that we recognize the wide range and sometimes contradictory effects on CECs that are elicited by these organisms. The diverse genes that are altered by gut microbes range from those involved in metabolism and signaling to those functioning in bacterial recognition and immune surveillance; most of these genes have not been linked to CRC development.

Notably, many of the studies that identified CRC-related genes or pathways that are modified by the gut microbiome were not designed to examine CRC-related effects specifically. For example, Kelly and colleagues [94] sought to identify genes altered by the gut microbiome that showed different patterns of H3K4 methylation in individuals with inflammatory bowel disease, but these genes also relate to CRC because similar biological processes are disrupted in the two diseases. Similar to the computational analysis conducted by Liang and colleagues [119], in which microarray data (from a single laboratory) were reanalyzed to look for lncRNA changes induced by the gut microbiome, or the meta-analysis conducted by Drewes and colleagues [7], in which combined data from several groups were reanalyzed through a single computational pipeline, studies examining the impact of the gut microbiome on the epigenome should be reanalyzed to probe for CRC-related alterations that were not explored in the original analyses. Such reanalyses would enhance our understanding of how frequently gut microbes induce epigenomic changes in genes that are related to CRC. There are clear technological hurdles that make this approach challenging. For example, the studies mentioned above utilize several different methods to probe the epigenome, hindering direct cross-comparisons. With ongoing computational advances, analytical pipelines continue to evolve and an expectation of standardized methods appears unlikely. Nonetheless, reanalysis of differing, often small, genomic or epigenomic datasets using a single computational approach may have value in discerning signals and generating new hypotheses for further testing [7].

Once CRC-related genes that are consistently altered by the gut microbiome are identified, we can begin to assess their role in tumor development more systematically. Studies by Donohoe and colleagues [85] exemplify how an AOM/DSS model of CRC can be used to explore the effect of gut microbial composition or organization on tumor development, with subsequent analysis of the CEC epigenome and genome changes that contribute to tumorigenesis. For example, colon tumors can be analyzed using many different techniques, including ChIP-seq, RNA-seq, DNase-seq, microarrays, and reduced representation bisulfite sequencing (RRBS). Studies by Wang and colleagues [63] typify how a mouse xenograft model can be used to investigate the mutagenic capacity and tumorigenic potential of specific microbes in vitro. As technology advances, we may soon be able to simulate gut microbiome–CEC interactions in vitro and to investigate the impact of modulating microbial communities in a xenograft model [120, 121]. Studies by O’Hagan and colleagues [122] illustrate how genetic mouse models of CRC can be used to examine changes in the epigenome of tumors that are induced directly by gut microbes. Furthermore, studies by Maiuri and colleagues [61] demonstrate how genetic mouse models of DNA damage pathways can be combined with genetic mouse models of CRC to determine whether specific microbes contribute to tumorigenesis through an accumulation of DNA mutations that would normally be repaired by well-characterized DNA damage repair pathways.

The microbiome community needs to marshal toward the utilization of diverse strategies to identify specific microbes, communities, and mechanisms governing genetic and epigenetic changes that can be targeted to enhance the screening, prevention, or treatment of CRC. Although recent studies have identified an association between both fungi and viruses in the gut and CRC development [123, 124], no specific impacts on CECs or their genomes or epigenomes have been described yet, providing additional opportunities for discovery.

## Conclusions and future directions

One clear goal moving forward is to explore how microbes can be used to better prevent CRC. Bacteria might act directly to impact CRC pathogenesis via the effect of one or more virulence factors on CECs, or indirectly via the production of secondary metabolites or the induction of immune changes in the mucosal environment; but how the immune system alters the genome or epigenome of CECs remains a gap in knowledge. As a result, if convincing data accrue that show that bacteria or bacterial communities directly influence colon carcinogenesis, then we may be able to target these bacteria for elimination from the colon via bacteriophage microbiome modulation or targeted antibiotics, or perhaps even develop protective vaccines against them or their virulence determinants. In this approach, the effect of gut microbes on the genome or epigenome of CECs could be utilized to monitor the effectiveness of the vaccine or bacterial elimination strategies, ensuring that other bacteria have not emerged to fill the niche left by the eliminated microbes and thus reduced the effectiveness of these prevention strategies.

Alternatively, bacteria- and gut microbe-induced genetic or epigenetic changes may also be included in approaches for early detection of CRC. Several studies have begun to assess the usefulness of including gut microbes in screening modalities for CRC but, to date, the performance metrics of such approaches limits their utility as clinically relevant screening strategies [9, 10, 125–127]. By contrast, screening strategies that utilize blood to detect mutated genes in cancer (including CRC) are rapidly developing into potentially viable tests [128], and strategies utilizing miRNAs and other epigenetic changes are being carefully considered [68, 129, 130]. It seems possible that the overall sensitivity and specificity of these screening modalities will be enhanced by including the specific gut microbes that contribute to the genetic or epigenetic changes being monitored, or by including gut microbes that are known to be associated with CRC in general.

Gut microbe-induced genetic or epigenetic changes may also inform the development of novel strategies for therapy. Bullman and colleagues [19] showed that *Fusobacterium* and other associated gut microbiome species were present in primary and metastatic human CRC. They also showed that primary CRC tumors were more readily transplanted into *nu*/*nu* mice if the tumor contained *Fusobacterium* species, and that the implanted tumors retained viable *F. nucleatum*, as well as other anaerobic species, including *B. fragilis*, for longer than 6 months [19]. In these experiments, the tumors grew more slowly when antibiotics were given to the mice after xenograft transplantation, but the authors did not identify the specific effect of *F. nucleatum* on tumors or determine whether addressing the downstream effect of bacterial presence (for example, stable epigenetic changes) might work synergistically with bacterial eradication to enhance tumor elimination. Overall, these data suggest that bacterial species contribute to tumor growth and metastasis, and that bacterial elimination might enhance a CRC treatment scheme, although it seems unlikely that bacterial elimination alone will halt disease progression given the clonal expansion of mutated CECs that defines CRC. The data produced by Yu and colleagues [117] complement the results of Bullman and colleagues [19] by showing that either removal of *F. nucleatum* or modulation of miRNA expression negated the consequences of bacterial presence in tumors, as tumor responsiveness to chemotherapy was potentially restored.

Overall, understanding and corralling knowledge of the microbiome to thwart disease and to augment disease therapy are towering cross-disciplinary goals. In a time when combination strategies are being implemented to address many diseases, both gut microbes and the genetic or epigenetic alterations that they induce are certain to add value to current targets for the prevention, detection, and treatment of CRC. As CRC is one of the diseases currently being studied most extensively in its connection to the microbiome, translational advancement in this field seems poised to spur progress in other microbiome-associated diseases.

## Abbreviations

AOM: Azoxymethane
BFT: *Bacteroides fragilis* toxin
CEC: Colon epithelial cell
CIN: Chromosomal instability
CRC: Colorectal cancer
DMR: Differentially methylated region
DSS: Dextran sodium sulfate
ETBF: Enterotoxigenic *Bacteroides fragilis*
H_2_S: Hydrogen sulfide
HDAC: Histone deacetylase
IESC: Intestinal epithelial stem cell
SCFA: Short chain fatty acid
